# The complete mitochondrial genome of *Cepola schlegelii* from the East China Sea

**DOI:** 10.1080/23802359.2022.2139158

**Published:** 2022-11-04

**Authors:** Panjiao Liang, Shouqiang Wang, Ye Lin, Li Wang, Linlin Zhao, Shenghao Liu

**Affiliations:** a School of Municipal and Environmental Engineering, Shenyang Jianzhu University, Shenyang, China; b Key Laboratory of Marine Eco-Environmental Science and Technology, First Institute of Oceanography, Ministry of Natural Resources, Qingdao, China

**Keywords:** Genomic DNA, mitochondria, evolutionary analysis, eupercaria, high-throughput sequencing

## Abstract

*Cepola schlegelii* (Bleeker 1854) belongs to the genus *Cepola* in the family Cepolidae and order Priacanthiformes. The complete mitochondrial genome of *C. schlegelii* was sequenced and analyzed by a high-throughput sequencing approach. The full length of the genome is 17,020 bp, including 13 protein-coding genes (PCGs), 22 transfer RNA genes (tRNAs), two ribosomal RNA genes (rRNAs), and a non-coding control region (D-loop). Phylogenetic analysis based on complete mitochondrial genomes revealed that *C. schlegelii* was most closely related to *Acanthocepola krusensternii*. The complete mitochondrial sequence of *C. schlegelii* will enrich the mitochondrial genome database and provide useful resources for population genetics and evolution analyses.

*Cepola schlegelii* is a Eupercaria species found in the South China Sea and the East China Sea (Liu 2000; Miyahara et al. 2002). Its body is orange-red when it is alive; its back is slightly darker, and its abdomen is slightly lighter. The fins and sides are unmarked, while a black circular spot is visible between the anterior jaw and maxilla. *C. schlegelii* is a bottom-dwelling fish that inhabits deep sandy or muddy waters. This fish typically digs a cave, hides within it, and stalks its prey by positioning itself near the cave with its heads up and tails down. Molecular genetic studies have not yet been conducted on *C. schlegelii*. This study provided the first complete mitochondrial genome of this species. Additionally, the evolutionary relationship of Eupercaria fishes was determined through phylogenetic analysis.

The sample was collected from the East China Sea (26°91’N,123°32’E) in October 2021 using the Agassiz trawl method. The voucher specimen (no. FIOSSC01) was stored at the First Institute of Oceanography, Ministry of Natural Resources of China (http://en.fio.org.cn/, Shenghao Liu, shliu@fio.org.cn). Species identification was carried out according to previously described methods (Wu and Zhong 2021). Genomic DNA was extracted from the muscle of a dorsal fin using a DNeasy Blood & Tissue Kit (Qiagen, Hilden, Germany). Briefly, 25 mg tissue was cut into small pieces and placed in a 1.5 mL microcentrifuge tube. 180 μL ATL buffer and 20 μL Proteinase K were added to the sample and mixed thoroughly by vortexing. The sample was incubated at 56 °C to make the tissue completely lysed. Then, 4 μL RNase A (100 mg/mL) was added to the sample and incubated for 2 min at room temperature (15–25 °C) to remove total RNA. 200 μL Buffer AL was added to the sample, and mix thoroughly by vortexing. Then, 200 μL ethanol (95%) was added and mixed thoroughly by vortexing. The sample mixture was transferred into the DNeasy mini spin column and centrifuge at 10,000 g for 1 min, and the genomic DNA was bound onto the DNeasy membrane. In order to thoroughly wash the genomic DNA, 500 μL Buffer AW1 and Buffer AW2 were added and centrifuged, respectively. The spin column was then air-dried for 3 min at room temperature. Finally, 200 μL Buffer AE was added onto the DNeasy membrane and incubated at room temperature for 2 min, and the genomic DNA was collected by centrifuge for 1 min at 10,000 g.

The mitochondrial genomic DNA was sequenced using the high-throughput sequencing platform MGISEQ-T7 (BGI Genomics, Shenzhen, China) with an insert size of 350 bp. The mitochondrial genome was assembled from clean data with GetOrganelle v1.7.5.3 (Freudenthal et al. 2020) according to the reference genome of *Acanthocepola krusensternii* (GenBank accession number: AP006812). The mitochondrial genome was then annotated using the MitoFish and MITOS databases (Bernt et al. 2013; Iwasaki et al. 2013). The mitochondrial genome assembly data were deposited in GenBank database from the National Center for Biotechnology Information (NCBI) with accession number ON110125.

The complete mitochondrial genome is 17,020 bp, containing 13 protein-coding genes (*PCGs*), 22 transport RNA genes (*tRNAs*), two ribosomal RNA genes (*rRNAs*), and one non-coding control region (D-loop) (Figure 1). The nucleotide composition consists of 26.49% A, 17.27% G, 29.09% C, and 27.15% T. The lengths of the 12S *rRNA* and 16S *rRNA* are 949 bp and 1,656 bp, respectively. The sizes of the 22 *tRNAs* range from 67 bp to 73 bp. *PCGs* begin with the normal ATG start codon, whereas *CO1* starts with GTG. Eight *PCGs* (*ND1*, *ND2*, *ND3*, *ND5*, *ATP8*, *ATP6*, *ND4L,* and *ND6*) terminate with a complete stop codon (ATG/T), whereas three *PCGs* (*CO2*, *CO3,* and *ND4*) terminate with an incomplete stop codon (T––). The light chain carries eight *tRNAs* (*tRNA-Glu, tRNA-Pro, tRNA-Gln, tRNA-Ala, tRNA-Asn, tRNA-Cys, tRNA-Tyr,* and *tRNA-Ser*) and *ND6*, while the heavy chain contains the remaining *tRNAs*.

**Figure 1.**
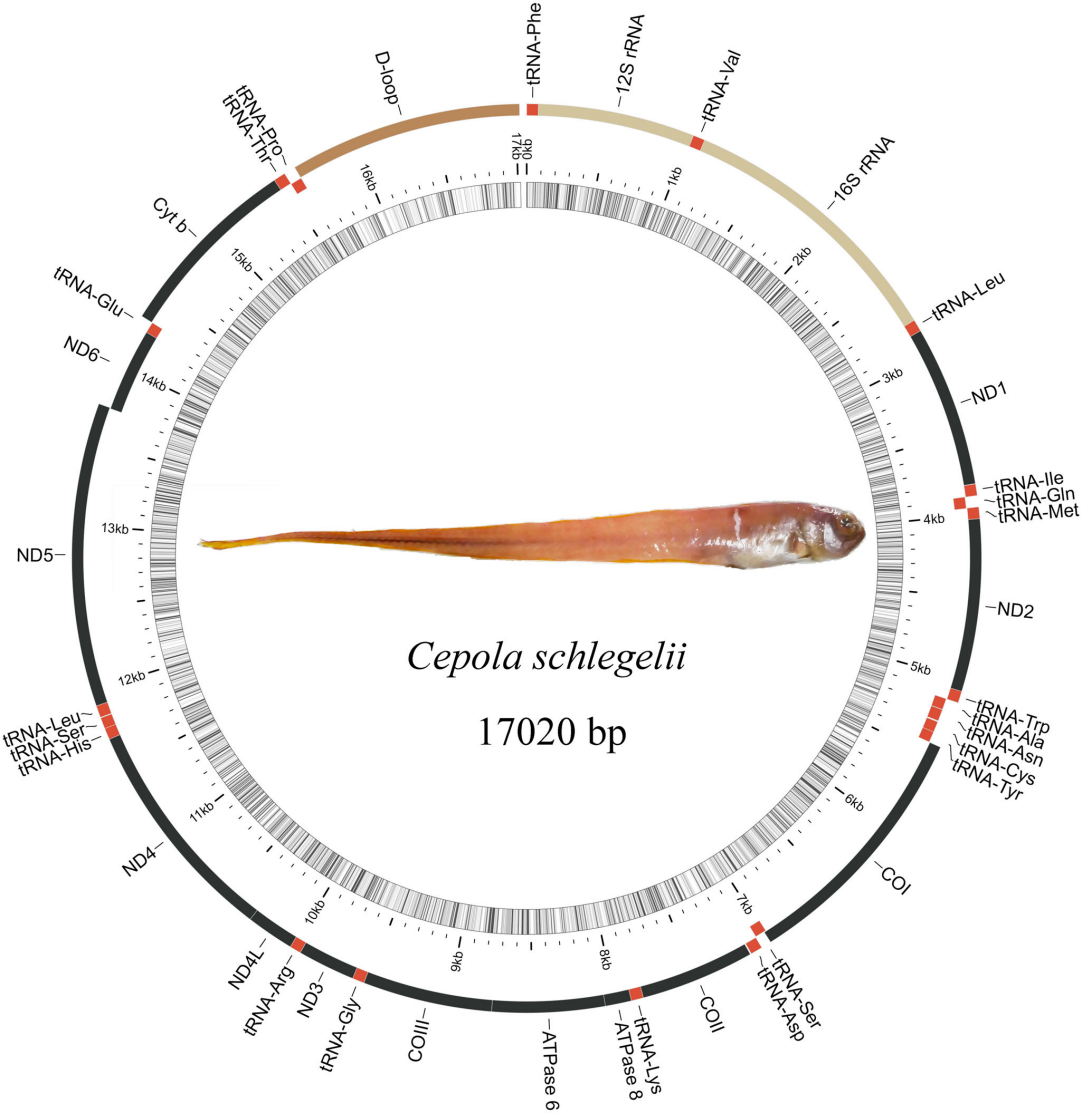
Mitogenome pattern map of *Cepola schlegelii.*

To investigate the evolutionary relationship of *C. schlegelii*, we constructed a phylogenetic tree using the maximum-likelihood (ML) method based on 13 protein-coding nucleotide sequences from the Eupercaria, Anabantaria and Carangaria mitogenomes. Multiple sequence alignment was conducted using MAFFT (Katoh and Standley 2013). Phylogenetic tree was constructed using IQ-TREE software (v1.6.12) from Nucleic acid sequences (with maximum-likelihood method, 1000 replicates, and TVM + F + R5 model; Nguyen et al. 2015). The results showed that *C. schlegelii* and *A. krusensternii* clustered together, forming the Cepolidae family branch. These two mitochondrial genomes are the only Cepolidae family members in the public database. In the phylogenetic tree, *C. schlegelii*, *A. krusensternii*, *Priacanthus macracanthus*, and *Priacanthus tayenus* constitute the priacanthiformes order. *Diplodus sargus*, *Dentex dentex*, and *Scolopsis vosmeri* cluster together and form the Spariformes order. Therefore, *C. schlegelii* is the closest relative to *A. krusensternii*, while *C. schlegelii* is far from other species (Figure 2).

**Figure 2.**
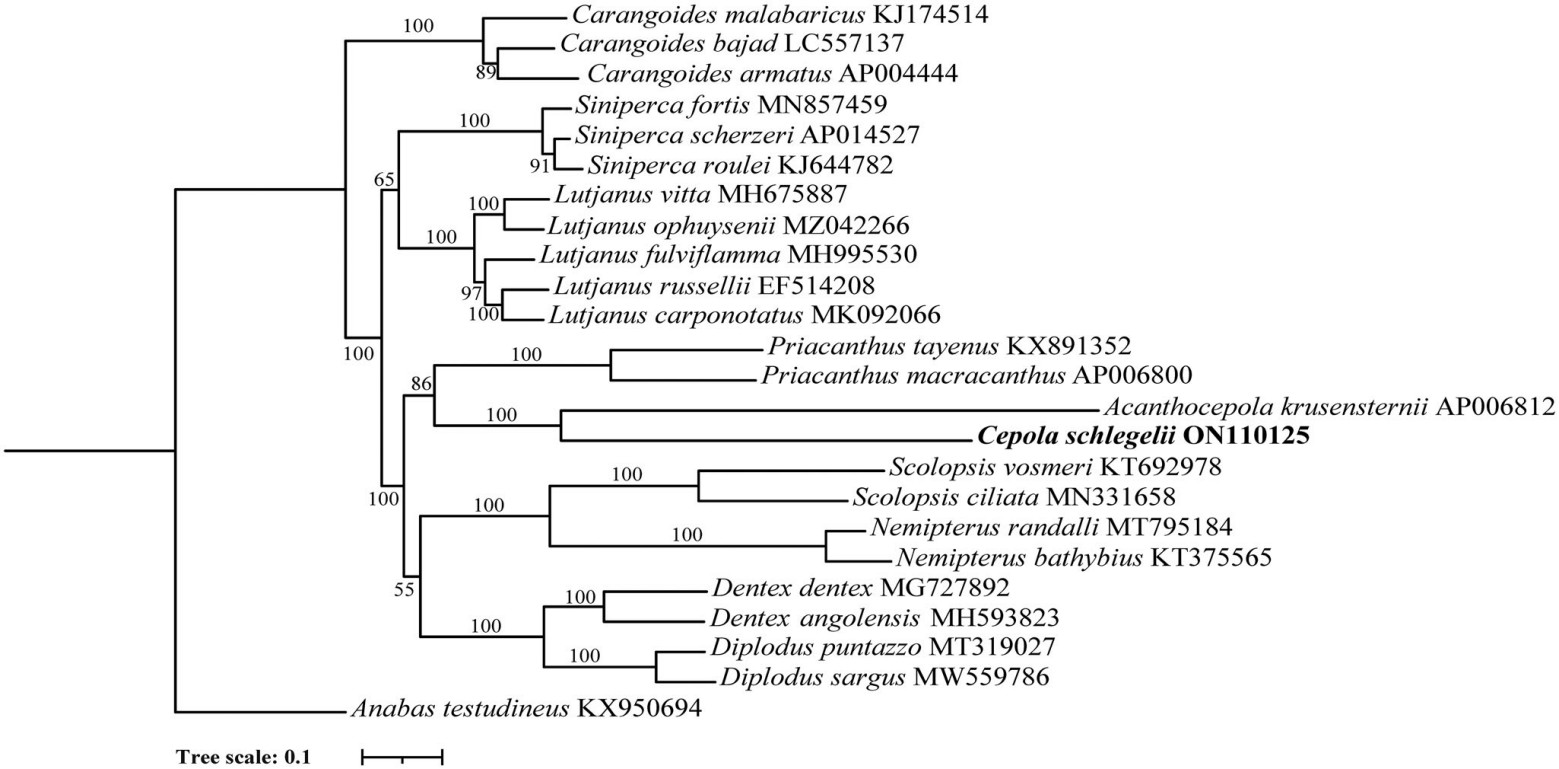
The maximum-likelihood (ML) phylogenetic tree indicated the relationships among *Cepola schlegelii* and other species in the *Eupercaria. Anabas testudineus* was used as the outgroup. Numbers on the nodes represent bootstrap values. GenBank accession numbers of each species are listed in the tree.

In conclusion, we presented the complete mitochondrial genome sequence of *C. schlegelii* and conducted a phylogenetic analysis of Eupercaria. The complete mitochondrial sequence will provide important information for population genetics studies and germplasm conservation.

## Data Availability

The complete mitochondrial genome assembly data were available in GenBank database under the accession number ON110125 (https://www.ncbi.nlm.nih.gov/nuccore/ON110125.1/). The high throughput sequencing reads for mitochondrial genome assembly have been deposited in the National Genomics Data Center (NGDC, https://ngdc.cncb.ac.cn) under the BioProject number PRJCA009031, BioSample number SAMC713165, and Genome Sequence Archive (GSA) databse with the accession number CRR458293 (https://ngdc.cncb.ac.cn/gsa/browse/CRA006611/CRR458293).
